# Supplementary material for: Chemical constituents and biological activities of endophytic fungi from Fagopyrum dibotrys
Source: PeerJ. 2024 Nov 18;12:e18529. doi: 10.7717/peerj.18529 (PMC11580677; doi:10.7717/peerj.18529)
Supplement: Supplemental Information 3 [file peerj-12-18529-s003.pdf]

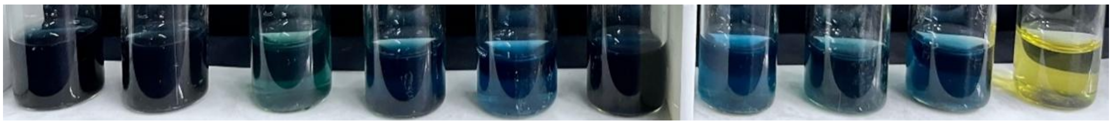

Figure S1. color reaction of the polyphenol-producing endophytic fungi

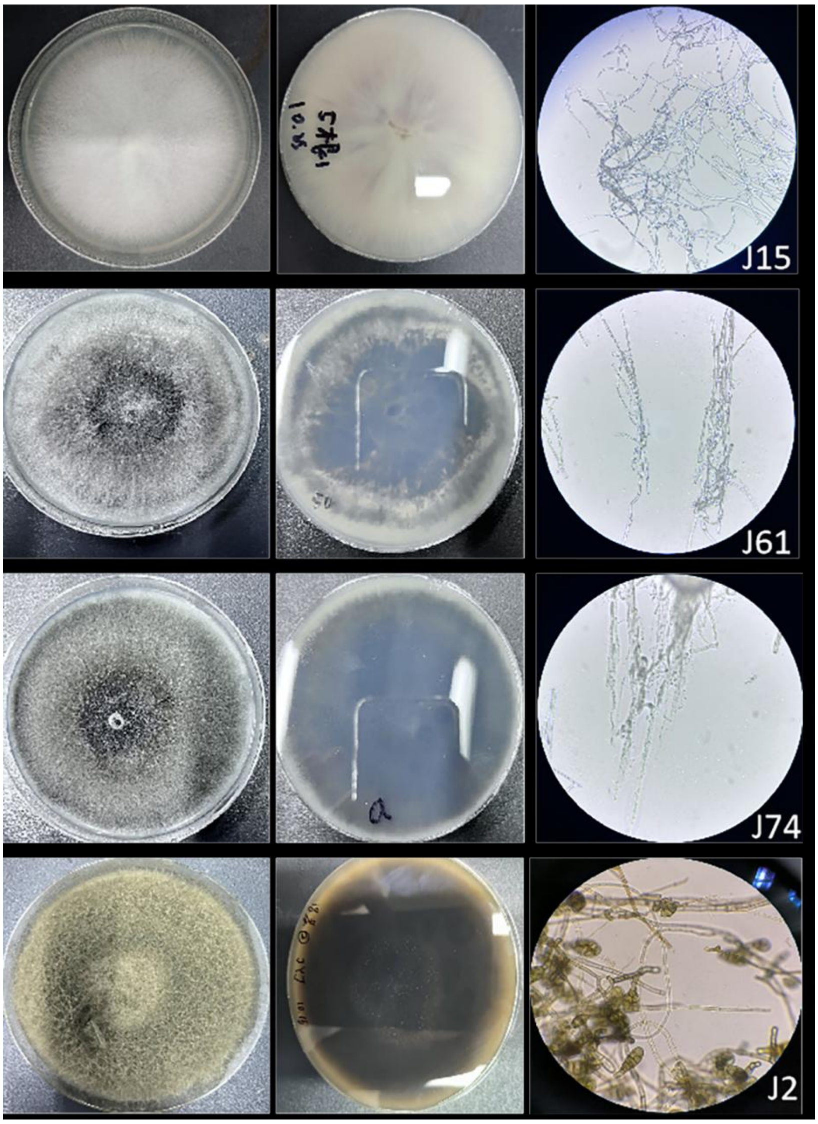

Figure S2. Microscopic and morphological characteristics images of endophytic fungi isolated from *Fagopyrum dibotrys*

Table S1. Morphological observation of endophytic fungi isolated from *Fagopyrum dibotrys*

Endophyte	colour	Morphology
J2	green	mycelium is green and the colony is convex and dry.
J15	White	mycelium is white and the colony is flat and dry.
J61	gray and white	air mycelia is brown, and the endophytic mycelia is black in center and white in margin.
J74	gray and black	air mycelium is grey and the colonies are flat and dry.

Table S2 Assessment of TPC and antioxidant activity of J2, J15, J61, J74 extracts.

**Extracts**	**TPC（mg GAE/g of extract）**	**IC_{50}（mg/mL）**			
		**ABTS radical**	**Hydroxyl radical**	**DPPH radical**	**Superoxide anion radical**
**J2**					
Vc	-	-	$0.07 \pm 0.05_{c}$	-	$0.26 \pm 0.02_{b}$
Ethyl acetate	$135.25 \pm 0.33_{a}$	$0.049 \pm 0.005_{c}$	$0.08 \pm 0.001_{c}$	$0.02048 \pm 0.009_{c}$	$0.28 \pm 0.005_{b}$
n-Butanol	$62.18 \pm 5.41_{b}$	$0.034 \pm 0.002_{c}$	$0.96 \pm 0.02_{b}$	$0.14368 \pm 0.004_{b}$	$1.54 \pm 0.004_{a}$
Chlorom	$42.72 \pm 4.39_{c}$	$1.568 \pm 0.002_{a}$	$0.15 \pm 0.06_{c}$	$0.3795 \pm 0.0025_{b}$	nd
Petroleum ether	$26.49 \pm 7.25_{d}$	$0.224 \pm 0.003_{b}$	$1.47 \pm 0.22_{a}$	$0.79028 \pm 0.004_{a}$	nd
**J15**					
Vc	-	-	$0.07 \pm 0.05_{d}$	-	$0.26 \pm 0.02_{b}$
Ethyl acetate	$92.74 \pm 4.68_{a}$	$0.051 \pm 0.0002_{d}$	$0.25 \pm 0.006_{c}$	$0.08 \pm 0.001_{b}$	$0.65 \pm 0.041_{a}$
n-Butanol	$67.39 \pm 6.81_{b}$	$0.406 \pm 0.006_{c}$	$0.54 \pm 0.03_{b}$	$0.16 \pm 0.021_{b}$	nd
Chloro	$14.11 \pm 5.47_{c}$	$1.751 \pm 0.0001_{a}$	$0.19 \pm 0.025_{c}$	nd	nd
Petroleum ether	$53.96 \pm 4.31_{b}$	$0.767 \pm 0.001_{b}$	$0.98 \pm 0.021_{a}$	$1.25 \pm 0.004_{a}$	nd
**J74**					
Vc	-	-	$0.07 \pm 0.05_{d}$	-	$0.26 \pm 0.02_{b}$
n-Butanol	$97.76 \pm 4.31_{a}$	$0.098 \pm 0.001_{b}$	$0.7 \pm 0.005_{b}$	$0.08 \pm 0.005_{c}$	$0.66 \pm 0.039_{b}$
Ethyl acetate	$65.80 \pm 7.25_{b}$	$0.596 \pm 0.011_{a}$	$0.51 \pm 0.047_{c}$	$0.61 \pm 0.003_{b}$	$1.56 \pm 0.028_{a}$
Chlorom	$52.89 \pm 5.11_{b}$	$0.058 \pm 0.015_{b}$	$0.33 \pm 0.035_{c}$	nd	nd
Petroleum ether	$13.75 \pm 5.25_{c}$	$0.847 \pm 0.002_{a}$	$0.84 \pm 0.004_{a}$	$1.62 \pm 0.001_{a}$	nd
**J61**					
Vc	-	-	$0.07 \pm 0.05_{d}$	-	$0.26 \pm 0.02_{b}$
Ethyl acetate	$129.64 \pm$	$0.073 \pm$	$0.57 \pm$	$0.01 \pm 0.032_{c}$	$0.23 \pm$

	4.28_a	0.003_c	0.005_b		0.006_b
n-Butanol	$68.90 \pm 5.81_b$	$0.089 \pm 0.013_c$	$0.43 \pm 0.038_b$	$0.06 \pm 0.012_c$	$1.24 \pm 0.043_a$
Chlorom	$47.40 \pm 3.39_c$	$0.672 \pm 0.012_b$	$0.25 \pm 0.007_c$	$0.15 \pm 0.004_c$	$1.89 \pm 0.024_a$
Petroleum ether	$28.96 \pm 6.55_d$	$1.18 \pm 0.013_a$	$0.77 \pm 0.003_a$	$0.32 \pm 0.005_a$	nd

a–d: significant differences between different groups ($p < 0.05$).

nd, not detected (result higher 3.00 mg/mL).

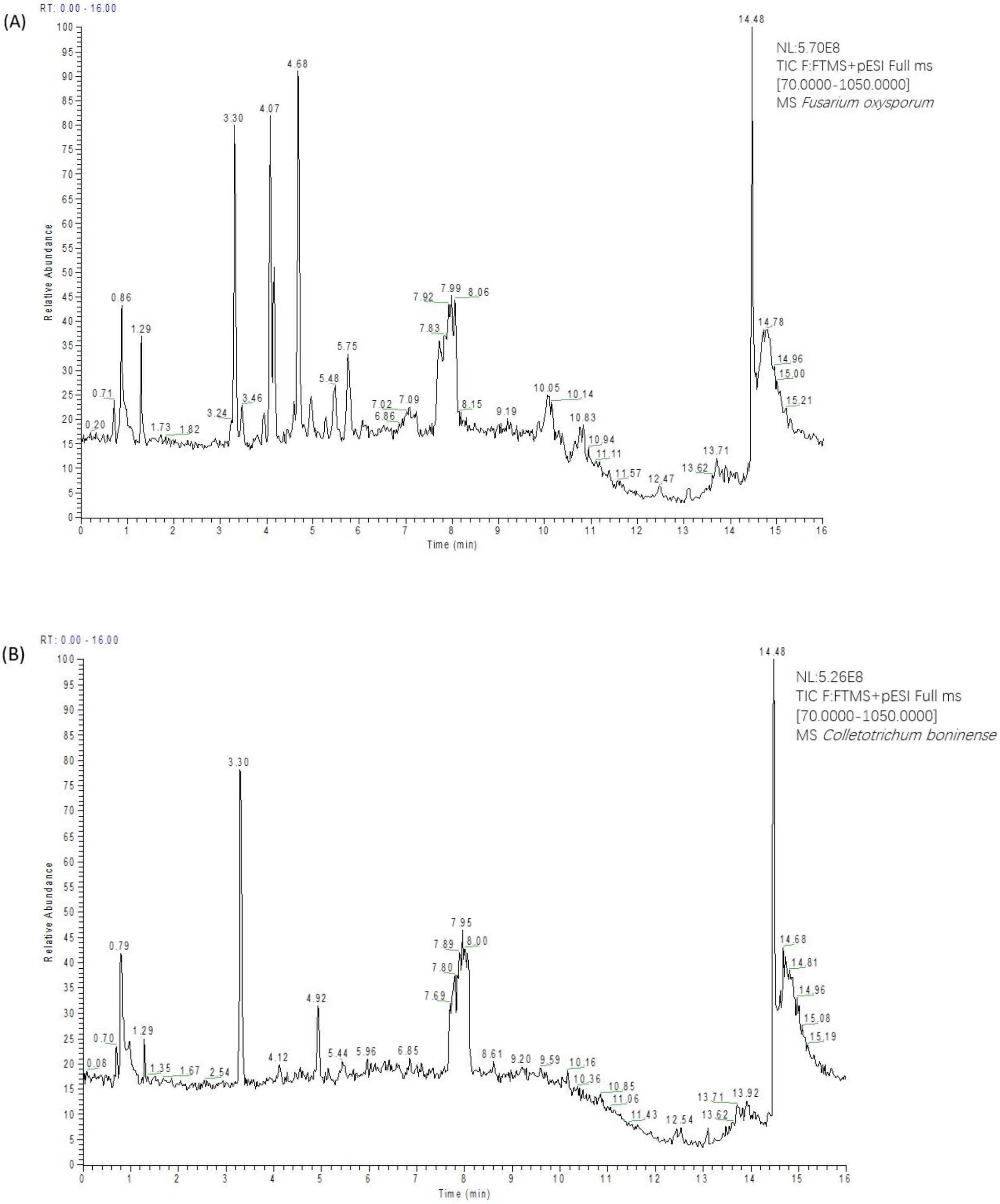
(A)
RT: 0.00 - 16.00
NL:5.70E8
TIC F:FTMS+pESI Full ms
[70.0000-1050.0000]
MS *Fusarium oxysporum*
Relative Abundance
Time (min)
14.48
4.68
3.30
4.07
0.86
1.29
7.92
7.99
8.06
7.83
5.75
5.48
14.78
14.96
15.00
15.21
10.05
10.14
10.83
10.94
11.11
11.57
12.47
13.62
13.71
0.71
0.20
1.73
1.82
3.24
3.46
7.02
7.09
6.86
8.15
9.19
(B)
RT: 0.00 - 16.00
NL:5.26E8
TIC F:FTMS+pESI Full ms
[70.0000-1050.0000]
MS *Colletotrichum boninense*
Relative Abundance
Time (min)
14.48
3.30
0.79
1.29
0.70
0.08
1.35
1.67
2.54
4.12
4.92
5.44
5.96
6.85
7.95
7.89
8.00
7.80
7.69
8.61
9.20
9.59
10.16
10.36
10.85
11.06
11.43
12.54
13.62
13.71
13.92
14.68
14.81
14.96
15.08
15.19

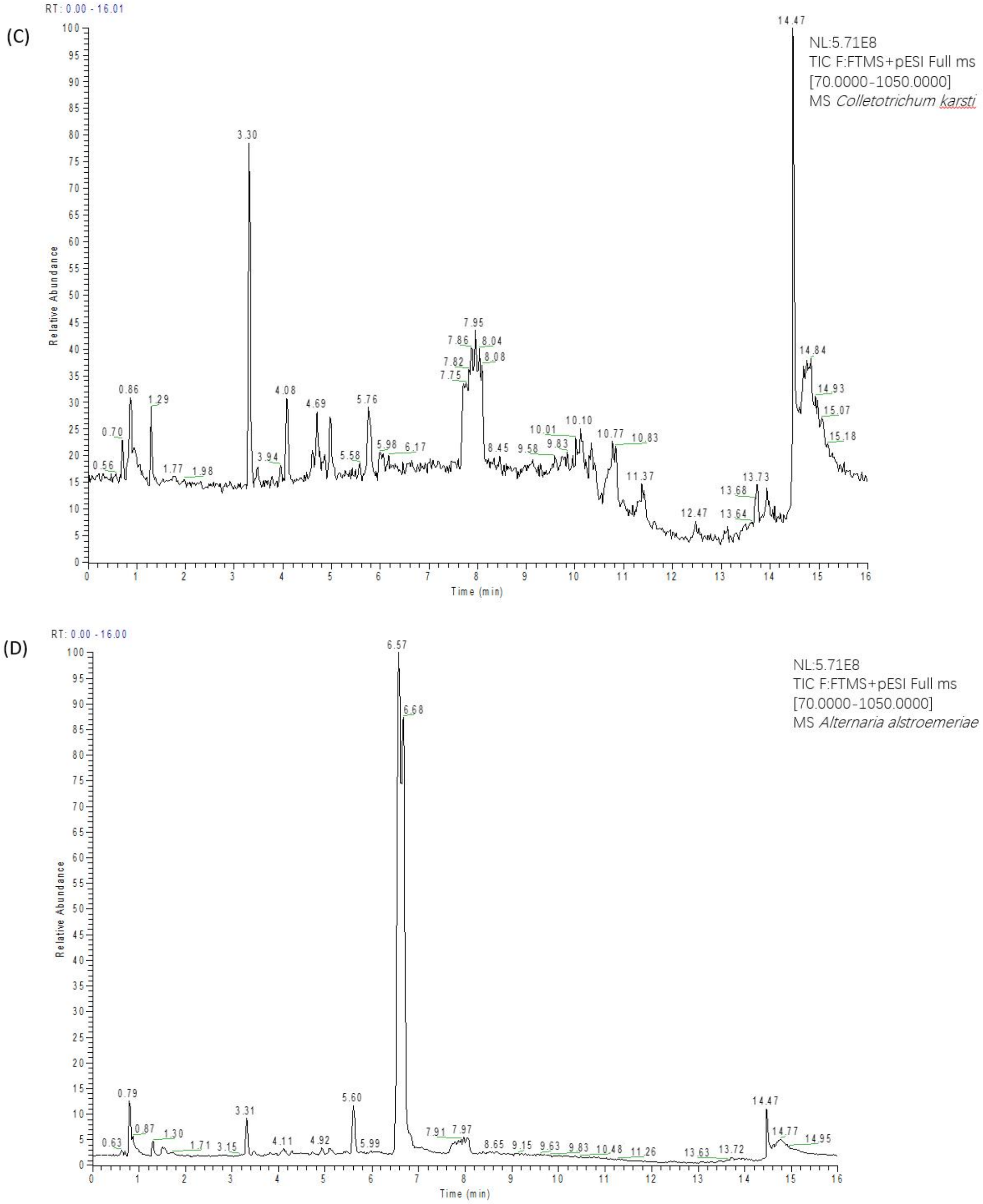

Figure S3. LC/MS chromatogram of fungal extracts. **(A)** LC/MS chromatogram of *Fusarium oxysporum* extracts; **(B)** LC/MS chromatogram of *Colletotrichum boninense* extracts; **(C)** LC/MS chromatogram of *Colletotrichum karsti* extracts; **(D)** LC/MS chromatogram of *Alternaria alstroemeriae* extracts
